# Single‐Segment Spinal Decompression and Fusion With Selective Nerve Root Block in Adult Degenerative Scoliosis: A Retrospective Comparative Study

**DOI:** 10.1111/os.70305

**Published:** 2026-04-02

**Authors:** Haoning Ma, Sizheng Zhan, Xiangsheng Tang, Ping Yi

**Affiliations:** ^1^ Department of Spine Surgery China‐Japan Friendship Hospital Beijing China

**Keywords:** adult degenerative scoliosis, posterior lumbar interbody fusion, selective nerve root block

## Abstract

**Objective:**

Adult degenerative scoliosis (ADS) presents a therapeutic dilemma between achieving adequate neural decompression and avoiding the morbidity associated with long‐segment fusion. This study aimed to compare clinical outcomes of patients diagnosed with ADS undergoing single‐segment posterior lumbar interbody fusion (PLIF) with selective nerve root block (SNRB) versus long‐segment PLIF.

**Methods:**

A retrospective cohort study was conducted of patients with ADS undergoing elective spinal fusion at a single medical center from July2019 to December 2021. Cohorts were divided into single‐segment PLIF with SNRB (SS‐PLIF+SNRB) or long‐segment (≥ 3) PLIF (LS‐PLIF) groups. The preoperative and postoperative spinal pelvic parameters were measured using X‐rays. Clinical symptoms were measured using the Oswestry Disability Index (ODI) and a visual analog scale (VAS). The patients' quality of life was evaluated using Short Form‐12. Length of stay, postoperative complications, and revision surgery were recorded. Continuous variables were analyzed using Student's *t*‐test or Mann–Whitney *U* test as appropriate, while categorical variables were compared using chi‐squared or Fisher's exact tests.

**Results:**

A total of 87 patients (32 single, 55 long) were included. There were no significant differences in age, gender, BMI, preoperative spinal pelvic parameters, or preoperative clinical symptoms between the groups (*p* > 0.05). Patients in the SS‐PLIF+SNRB group had a shorter hospital stay (*p* < 0.01) and a lower revision surgery rate (*p* < 0.01). No significant differences were noted in postoperative complications (*p* > 0.05). Patients in the SS‐PLIF+SNRB group had better improvements in ODI and SF‐12 scores 6 months after surgeries, and no significant differences were found in all clinical outcomes at the final follow‐up.

**Conclusion:**

Single‐segment PLIF with SNRB can achieve the same efficacy as a long‐segment PLIF for treating ADS. Single‐segment PLIF with SNRB in appropriately selected patients may provide satisfactory improvements in ODI, SF‐12, and VAS leg score and mitigate hospital length of stay and revision surgery rates.

## Graphical abstract

Single‐segment PLIF *with preoperative SNRB* achieves equivalent clinical outcomes to long‐segment fusion in adult degenerative scoliosis. This approach significantly reduces hospitalization duration and revision rates in appropriately selected patients.

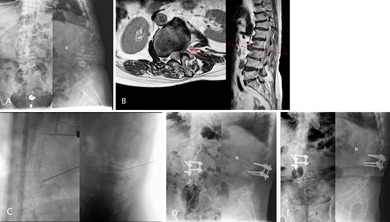

## 1 Introduction

Adult degenerative scoliosis (ADS), defined as a spinal deformity with a coronal Cobb angle exceeding 10° developing postadulthood, typically manifests around the fifth to sixth decade of life and progresses with advancing age [1]. Characterized by back pain, radicular symptoms, neurogenic claudication, and progressive coronal/sagittal imbalance, ADS poses a significant burden in an aging population due to its association with asymmetric intervertebral disk degeneration, facet arthropathy, and ligamentous laxity [2]. Surgical intervention is warranted in patients manifesting severe symptomatology refractory to conservative management strategies [3].

The decompression followed by posterior lumbar interbody fusion is the conventional technique to treat ADS [4]. The primary objective of this procedure is to achieve complete decompression of neural elements while simultaneously restoring sagittal and coronal spinal equilibrium through biomechanical realignment. Most patients with ADS are elderly with a broad array of medical comorbidities, imposing a high risk of postoperative complications. Short‐segment spinal fusion demonstrates distinct advantages, including reduced surgical expenses, minimized perioperative risks of complications and morbidity, and accelerated postoperative mobilization [5]. Long‐term follow‐up studies (48 months) demonstrate sustained clinical improvement in lumbar spinal stenosis cohorts managed with short‐segment decompression and fusion, though progressive loss of radiographic correction and limited deformity amelioration are inherent limitations [6]. Conversely, while providing superior biomechanical correction, long‐segment spinal fusion is associated with increased iatrogenic paraspinal muscle injury and significant blood loss, which collectively impede early and rapid rehabilitation [7, 8, 9]. In patients with ADS manifesting predominant radicular pain or neurogenic claudication in the lower extremities, the absence of low back pain induced by severe sagittal/coronal imbalance may obviate the necessity for long‐segment spinal fusion [10, 11].

Selective nerve root block (SNRB) is a minimally invasive procedure that involves the targeted puncture, localization, and administration of diagnostic/therapeutic agents to suspected pathological nerve roots (cervical, thoracic, lumbosacral regions) under multimodal imaging guidance (fluoroscopy, computed tomography, or ultrasonography), following systematic clinical assessment [12, 13]. In lumbar spine surgery, SNRB is of great significance in clarifying the responsible segments, reducing the surgical segments, and predicting the postoperative effect [14, 15]. The use of diagnostic SNRB in treating ADS has been rarely reported. Patients with positive SNRB responses can accurately pinpoint single‐segment PLIF targets, enabling focused surgery while avoiding extended decompression and fusion.

Therefore, the purposes of this study were:

1. To compare the clinical and radiographic outcomes between single‐segment PLIF with SNRB and long‐segment PLIF in patients with ADS;
2. To evaluate whether SNRB can facilitate accurate identification of symptomatic segments and enable limited fusion without compromising mid‐term outcomes;
3. To explore potential surgical indications for single‐segment PLIF with SNRB based on symptom patterns and deformity severity.

## 2 Materials and Methods

After approval from the Ethics Committee of China‐Japan Friendship Hospital (Approval No. 2024‐KY‐263), a retrospective review of all patients who underwent spinal fusion for symptomatic degenerative scoliosis from July 2019 to December 2021 at a single medical center was performed. All patients meet the following surgical indications: (1) low back and leg pain that progressively intensifies, severely impairing daily life and proving unresponsive to nonsurgical therapeutic interventions; (2) accompanied by lumbar spinal stenosis or refractory nerve root pain with neurological deficits, manifesting as intermittent claudication; (3) progressive exacerbation of scoliosis, with scoliosis progression > 10°; (4) no surgical contraindications were found in preoperative evaluation.

Inclusion criteria are as follows: (1) patients with more than 10° of Cobb angle; (2) aged more than 40 years; and (3) a completion of more than 2 years follow‐up.

Exclusion criteria were as follows: (1) Idiopathic or congenital scoliosis; (2) Patients with previous decompression or fusion surgery, or those treated for trauma, tumor, or infection. (3) Severe osteoporosis (T‐score < −3.5); (4) Severe neurological deficits as paraplegia; (5) Severe sagittal imbalance needed correction as SVA > 50 mm or PI‐LL > 10°.

A total of 87 patients were allocated into two treatment groups: the single‐segment posterior lumbar interbody fusion with selective nerve root block (SS‐PLIF+SNRB) group and the long‐segment posterior lumbar interbody fusion (LS‐PLIF) group. Demographic data including age, body mass index (BMI), gender, bone mineral density (BMD), and fused segments, hospital length of stay were obtained. BMD in patients was expressed as T‐scores, where the T‐score for each individual was calculated as the mean of T‐scores obtained from dual‐energy X‐ray absorptiometry (DXA) scans of the lumbar spine. Spinal pelvic parameters measurements were defined with neural standing X‐ray images. Pelvic tilt (PT) was defined by the angle between the line connecting the midpoint of the S1‐endplate to the axis of the femoral heads and the vertical plane. Pelvic incidence (PI) was defined as the angle perpendicular to the sacral endplate at its midpoint and the line connecting this point to the axes of the femoral heads. The C7 sagittal vertical axis (SVA) was defined by the horizontal distance from the superior posterior end of the upper sacral endplate to the C7 plumbline. The scoliosis Cobb angle was also measured. The lumbar function was evaluated using the Oswestry Disability Index (ODI), and the degree of low back pain and lower limb pain was evaluated using a visual analog scale (VAS). A △Value was calculated for each clinical outcome and was defined as preoperative minus the postoperative value. The patients' quality of life was evaluated using Short Form‐12 Physical Component score (PCS‐12) and Short Form‐12 Mental Component Score (MCS‐12).

### 2.1 Surgical Procedures

In the SS‐PLIF+SNRB group, all patients underwent diagnostic therapy with SNRB to identify the responsible segment prior to open surgery [16]. The injections were performed under fluoroscopy while the patient lay prone. The entry point is generally selected 10–12 cm away from the posterior median line. After local anesthesia with 1% lidocaine, a 15‐cm, 20‐gage spinal needle was advanced and positioned near the target nerve root. The target point was around the neural foramen. The needle position was checked by fluoroscopy and the pain provocation with nerve root irritation by needle contact was carefully carried out. The pain induced by the stimulation should correspond to the radiating pain area of the patient's lower extremities. Then, 0.5 mL of 1% lidocaine was injected. A VAS leg score improvement of more than 50% was considered a positive result for SNRB. Then, within the next 2 days, a single‐segment PLIF was performed.

In the PLIF procedure, the patients were positioned prone. An incision was made in the middle of the posterior square of the back. Paraspinal muscles were dissected layer by layer to expose the lamina and articular process. Decompression was performed in the only segment same to SNRB in the SS‐PLIF+SNRB group, while in multiple segments based on symptoms and images in the LS‐PLIF group. Interbody fusion was performed with cages filled with autograft from a laminectomy. Fixation was performed with rods and pedicle screws. Several kinds of posterior osteotomies were performed, such as Smith‐Petersen osteotomy or Ponte osteotomy, to correct the deformity and rebalance the spine in the LS‐PLIF group.

### 2.2 Data Analysis

Statistical analysis was performed using SPSS version 27.0.0 (IBM Corp., Armonk, New York, USA). Normality of samples was determined using the Shapiro–Wilk test. Comparison of means for continuous variables between groups was performed using a Student *T*‐test, and a Mann–Whitney *U*‐test was used to compare means for nonparametric variables and nonnormal distributions. Preoperative and postoperative variables for the same patients were compared using a paired Student *T*‐test, whereas a Wilcoxon signed‐rank test was used for nonparametric variables. Categorical data was compared using Pearson *χ* ^2^ and Fisher exact tests as appropriate. A probability (*p*) value of < 0.05 was considered statistically significant.

## 3 Results

### 3.1 Patient Demographics and Surgical Characteristics

This study included 87 patients, of whom 32 underwent single‐segment PLIF with SNRB whereas 55 underwent long‐segment (≥ 3) PLIF. There were no significant differences found in age (single, 65.4 ± 7.57 years vs. long, 63.5 ± 5.23 years; *p* = 0.171), sex (single, 37.5% male vs. long, 43.6% male; *p* = 0.575), and BMI (single, 24.3 ± 5.76 vs. long, 26.7 ± 6.23; *p* = 0.079). Radiculopathy was more prevalent among patients undergoing single‐segment PLIF with SNRB than in the LS‐PLIF group. Significantly prolonged hospital stays were observed in patients undergoing long‐segment PLIF surgeries (single, 6.48 ± 2.29 days vs. long, 9.31 ± 5.25 days; *p* < 0.05). No significant differences in total postoperative complications were noted between groups (short, 6.25% vs. long, 10.9%; *p* = 0.468). The primary indication for revision surgery in both groups was progressive degeneration of adjacent segments (single, 3.13%; long, 10.9%). The second reason for revision surgery in the LS‐PLIF group was pseudarthrosis (9.09%), followed by adjacent level vertebral fracture (5.45%), and hardware complications (3.64%). While in the SS‐PLIF+SNRB group, deformity progression (3.13%) was recorded as the other reason for revision surgery (Table 1).

**TABLE 1:** Patient demographics and operative data.

	SS‐PLIF+SNRB (*n* = 32)	LS‐PLIF (*n* = 55)	*p* ^a^, ^b^
Age (years)	65.4 ± 7.57	63.5 ± 5.23	0.171
Sex			
Male	12 (37.5)	24 (43.6)	0.575
Female	20 (62.5)	31 (56.4)	
Body mass index (kg/m^2^)	24.3 ± 5.76	26.7 ± 6.23	0.079
Fused segments (*n*)	1	5.2 ± 2.4 (3–8)	
BMD (T‐score)	−1.25 ± 1.38	−1.76 ± 1.86	0.18
Chief complaints			
Low back pain	5 (15.6)	17 (30.9)	
Radiculopathy	9 (28.1)	12 (21.8)	
Mixed	18 (56.3)	26 (47.3)	
Length of stay (days)	6.48 ± 2.29	9.31 ± 5.25	0.005^c^
Postoperative complications			
Total complications	2 (6.25)	6 (10.9)	0.468
Epidural hematoma	1	1	
Radiculitis	1	2	
Cerebrospinal fluid leakage	0	2	
Wound infection	0	1	
Revision surgery	2 (6.25)	13 (24.07)	0.034^c^
Adjacent segment disease	1 (3.13)	3 (5.45)	
Pseudarthrosis	0	5 (9.09)	
Hardware complications	0	2 (3.64)	
Fracture	0	3 (5.45)	
Deformity progression	1 (3.13)	0	

^a^ Independent‐samples *t*‐test or Mann–Whitney *U* test for age, body mass index and length of stay.

^b^ Pearson *χ* ^2^ or Fisher exact test for sex and postoperative complications.

^c^ Significance level established at *p* < 0.05.

### 3.2 Patients' Spinal Pelvic Parameters and Clinical Outcomes

There were no significant differences in all spinal pelvic parameters between the two groups preoperatively. The Cobb angles of the SS‐PLIF+SNRB group were (25.68 ± 12.57)° and (21.27 ± 13.18)° while the Cobb angles of the LS‐PLIF group were (25.63 ± 12.16)° and (14.33 ± 7.24)° respectively before and after the surgery. A significant improvement was shown in Cobb angle in the LS‐PLIF group (*p* < 0.05). Besides, greater corrections were also found in LL (preoperative (34.48 ± 5.52)° vs. postoperative (37.36 ± 7.28)°) and PT (preoperative (25.23 ± 7.91)° vs. postoperative (21.15 ± 5.28)°) in the LS‐PLIF group (*p* < 0.05). PI and SVA showed no significant differences before and after surgery in both groups (*p* > 0.05) (Table 2).

**TABLE 2:** Patients' spinal pelvic parameters.

		Pre‐op	Post‐op	*p* ^a^
PI (°)	SS‐PLIF+SNRB	48.35 ± 5.54	46.87 ± 9.15	0.437
	LS‐PLIF	46.31 ± 8.64	47.90 ± 7.34	0.431
	*p* value^b^	0.234	0.566	
LL (°)	SS‐PLIF+SNRB	35.86 ± 8.14	36.67 ± 6.36	0.659
	LS‐PLIF	34.48 ± 5.52	37.36 ± 7.28	0.021^c^
	*p* value^b^	0.350	0.657	
PT (°)	SS‐PLIF+SNRB	23.68 ± 6.25	21.75 ± 7.33	0.261
	LS‐PLIF	25.23 ± 7.91	21.15 ± 5.28	0.002^c^
	*p* value^b^	0.345	0.660	
SVA (cm)	SS‐PLIF+SNRB	3.58 ± 3.87	3.27 ± 3.86	0.748
	LS‐PLIF	4.19 ± 4.83	3.97 ± 4.12	0.798
	*p* value^b^	0.544	0.437	
Cobb (°)	SS‐PLIF+SNRB	25.68 ± 12.57	21.27 ± 13.18	0.176
	LS‐PLIF	25.63 ± 12.16	14.33 ± 7.24	< 0.001^c^
	*p* value^b^	0.985	< 0.001^c^	

^a^ Paired‐sample *t*‐test or Wilcoxon rank test comparing preoperative and postoperative values.

^b^ Independent‐samples *t*‐test or Mann–Whitney *U* test comparing SS‐PLIF+SNRB and LS‐PLIF groups.

^c^ Significance level established at *p* < 0.05.

Preoperative ODI and VAS back scores were significantly higher in the LS‐PLIF group (single 40.2 ± 7.5, vs. long 43.5 ± 7.3, *p* = 0.047, in ODI; single 5.47 ± 2.66, vs. long 6.73 ± 2.23, *p* = 0.020, in VAS back), while preoperative VAS leg scores were significantly higher in the SS‐PLIF+SNRB group (single 7.13 ± 2.84, vs. long 5.27 ± 2.49, *p* = 0.002). No significant differences were found in MCS‐12 or PCS‐12 between the two groups (single 47.6 ± 9.4, vs. long 51.3 ± 5.56, *p* = 0.064, in MCS‐12; single 31.7 ± 6.62, vs. long 32.6 ± 8.77, *p* = 0.616, in PCS‐12) preoperatively. At the follow‐up time point of 6 months after the surgery, patients showed statistically significant improvements in all clinical outcomes except for ODI (preoperative 43.5 ± 7.3, vs. 6 months postoperative 39.3 ± 14.77, *p* = 0.061), MCS‐12 scores (preoperative 51.3 ± 5.56, vs. 6 months postoperative 49.3 ± 9.2; *p* = 0.170) and PCS‐12 (preoperative 32.6 ± 8.77, vs. 6 months postoperative 34.1 ± 9.56; *p* = 0.412) in the LS‐PLIF group, which did not improve significantly. The MCS‐12 and PCS‐12 scores were higher in the SS‐PLIF+SNRB group compared with the LS‐PLIF group (single 53.4 ± 8.4, vs. long 49.3 ± 9.2, *p* = 0.042 in MCS‐12; single 38.5 ± 10.3, vs. long 34.1 ± 9.56, *p* = 0.047 in PCS‐12). There were no significant differences in VAS leg or VAS back scores between the two groups 6 months after surgeries (*p* > 0.05). At the final follow‐up, significant improvements were found in all clinical outcomes (*p* < 0.05), and there were no significant differences between the two groups (*p* > 0.05) (Table 3).

**TABLE 3:** Patients' clinical outcomes.

		Pre‐op	6 months	*p* ^a^	Final follow‐up	*p* ^a^
ODI (%)	SS‐PLIF+SNRB	40.2 ± 7.5	32.8 ± 9.3	< 0.001^b^	23.5 ± 11.5	< 0.001^b^
	LS‐PLIF	43.5 ± 7.3	39.3 ± 14.77	0.061	28.4 ± 13.7	< 0.001^b^
	*p* value^c^	0.047^b^	0.028^b^		0.092	
VAS leg	SS‐PLIF+SNRB	7.13 ± 2.84	3.85 ± 2.67	< 0.001^b^	2.66 ± 2.53	< 0.001^b^
	LS‐PLIF	5.27 ± 2.49	3.68 ± 2.53	< 0.001^b^	2.48 ± 2.32	< 0.001^b^
	*p* value^c^	0.002^b^	0.768		0.737	
VAS back	SS‐PLIF+SNRB	5.47 ± 2.66	3.52 ± 2.37	< 0.001^b^	3.41 ± 2.68	< 0.001^b^
	LS‐PLIF	6.73 ± 2.23	3.56 ± 2.84	< 0.001^b^	3.75 ± 2.39	< 0.001^b^
	*p* value^c^	0.020^b^	0.947		0.542	
MCS‐12	SS‐PLIF+SNRB	47.6 ± 9.4	53.4 ± 8.4	0.012^b^	53.7 ± 9.3	0.013^b^
	LS‐PLIF	51.3 ± 5.56	49.3 ± 9.2	0.170	54.8 ± 8.4	0.011^b^
	*p* value^c^	0.064	0.042^b^		0.572	
PCS‐12	SS‐PLIF+SNRB	31.7 ± 6.62	38.5 ± 10.3	0.003^b^	37.9 ± 9.6	0.004^b^
	LS‐PLIF	32.6 ± 8.77	34.1 ± 9.56	0.412	36.4 ± 9.4	0.031^b^
	*p* value^c^	0.616	0.047^b^		0.478	

^a^ Paired‐sample *t*‐test or Wilcoxon rank test comparing preoperative and postoperative values.

^b^ Significance level established at *p* < 0.05.

^c^ Independent‐samples *t*‐test or Mann–Whitney *U* test comparing SS‐PLIF+SNRB and LS‐PLIF groups.

### 3.3 Subgroup Analysis Based on Cobb Angle

A subgroup analysis was conducted based on a preoperative Cobb angle threshold of 25°. Within the Cobb angle ≤ 25° cohort, the SS‐PLIF+SNRB group (*n* = 18) demonstrated a significantly greater improvement in VAS leg score compared to the LS‐PLIF group (*n* = 21) (5.63 ± 2.4 vs. 3.67 ± 2.9, *p* = 0.028). No significant differences were found between the two groups in the improvements of ODI (15.3 ± 4.5 vs. 16.8 ± 3.2, *p* = 0.23) or VAS back score (2.35 ± 1.64 vs. 2.86 ± 1.57, *p* = 0.32). The LS‐PLIF group showed a greater correction in Cobb angle (12.35° ± 7.74° vs. 6.34° ± 3.86°, *p* = 0.004).

In the Cobb angle > 25° cohort, the SS‐PLIF+SNRB group (*n* = 14) again exhibited a superior improvement in VAS leg score relative to the LS‐PLIF group (*n* = 34) (5.72 ± 3.48 vs. 3.45 ± 1.43, *p* = 0.002). However, in this cohort, the improvement in VAS back score was significantly greater in the LS‐PLIF group (*n* = 34) (3.78 ± 2.24 vs. 2.34 ± 1.86, *p* = 0.039). The improvement in ODI was comparable between the groups (17.3 ± 6.4 vs. 16.9 ± 7.3, *p* = 0.86). The LS‐PLIF group achieved a significantly larger Cobb angle correction (20.79° ± 5.26° vs. 7.54° ± 3.48°, *p* < 0.001) (Table 4).

**TABLE 4:** Subgroup analysis by Cobb angle.

	SS‐PLIF+SNRB cobb ≤ 25° (*n* = 18)	LS‐PLIF cobb ≤ 25° (*n* = 21)	*p* ^a^	SS‐PLIF+SNRB cobb > 25° (*n* = 14)	LS‐PLIF cobb > 25° (*n* = 34)	*p* ^a^
ΔODI (%)	15.3 ± 4.5	16.8 ± 3.2	0.23	17.3 ± 6.4	16.9 ± 7.3	0.86
ΔVAS leg	5.63 ± 2.4	3.67 ± 2.9	0.028^b^	5.72 ± 3.48	3.45 ± 1.43	0.002^b^
ΔVAS back	2.35 ± 1.64	2.86 ± 1.57	0.32	2.34 ± 1.86	3.78 ± 2.24	0.039^b^
ΔCobb (°)	6.34 ± 3.86	12.35 ± 7.74	0.004^b^	7.54 ± 3.48	20.79 ± 5.26	< 0.001^b^

^a^ Independent‐samples *t*‐test or Mann–Whitney *U* test comparing SS‐PLIF+SNRB and LS‐PLIF groups.

^b^ Significance level established at *p* < 0.05.

### 3.4 Subgroup Analysis of Clinical Outcomes Based on Bone Mineral Density in the SS‐PLIF+SNRB Group

In the subgroup analysis, 24 patients were classified as nonosteoporotic and 8 as osteoporotic. Patients in the osteoporosis subgroup were significantly older than those in the nonosteoporosis subgroup, which is consistent with the age‐related nature of bone loss (osteoporosis 69.5 ± 3.74, vs. nonosteoporosis 63.2 ± 4.33, *p* < 0.05). Both groups showed postoperative improvement in all clinical outcomes. However, there were no statistically significant differences between groups in the changes of ODI, VAS leg, or VAS back (Table 5).

**TABLE 5:** Subgroup analysis of clinical outcomes based on bone mineral density in the SS‐PLIF+SNRB group.

	Nonosteoporosis (*T* > −2.5)	Osteoporosis (−3.5 ≤ T ≤ −2.5)	*p* ^a^
No. of patients	*n* = 24	*n* = 8	—
Age (years)	63.2 ± 4.33	69.5 ± 3.74	0.001^b^
ΔODI (%)	17.2 ± 2.7	15.3 ± 2.4	0.09
ΔVAS leg	5.45 ± 2.7	5.28 ± 1.7	0.86
ΔVAS back	2.53 ± 1.46	2.24 ± 1.43	0.62

^a^ Independent‐samples *t*‐test or Mann–Whitney *U* test comparing nonosteoporosis and osteoporosis groups.

^b^ Significance level established at *p* < 0.05.

## 4 Discussion

ADS is associated with sagittal and coronal plane malalignment caused by asymmetric intervertebral disk degeneration and facet joint degeneration [17]. While all patients in the cohort exhibited coronal imbalance, a significant number presented with acute clinical manifestations linked to localized neural compression sites. Although local decompression effectively alleviates radicular symptoms, it may predispose to iatrogenic spinal instability. Most symptomatic degenerative scoliosis patients require spinal fusion combined with curvature correction alongside nerve decompression surgery. Long‐segment spinal internal fixation and fusion surgeries are often accompanied by a relatively high incidence of complications [8, 9, 18]. Contemporary clinical strategies increasingly favor segmental fusion techniques focused on symptomatic spinal levels to mitigate these iatrogenic risks [19]. When patients present with radicular pain or neuropathic intermittent claudication as their chief complaint, the therapeutic goal is to relieve the compression on the nerve; the correction of scoliosis seems to be secondary. Short‐segment instrumented fusion can be used as a feasible alternative to traditional long‐segment surgery in patients with Cobb angles less than 20° and with mild rotational deformity according to some clinical researches [20].

As an independent diagnostic method, SNRB was first reported by Macnab in 1971 [21]. Fluoroscopy‐guided SNRB involved contrast‐enhanced angiography of the targeted nerve root followed by lidocaine injection for diagnostic blockade, establishing its clinical utility in preoperative evaluation of patients with equivocal imaging findings (e.g., negative/mild MRI changes) or anatomically ambiguous lesion localization. For patients presenting multisegmental degenerative diseases of the spine, SNRB is helpful in identifying the responsible segments and the affected nerve roots, reducing surgical trauma, and is of great significance for minimally invasive and precise treatment [22]. Positive SNRB findings may enable surgical planning from short‐segment to single‐segment. The purpose of this study is to determine whether the treatment of ADS with single‐segment PLIF with SNRB can improve clinical outcomes compared with that of patients undergoing long‐segment PLIF.

### 4.1 Clinical Outcomes

All the patient‐reported outcomes of both groups including VAS, ODI, MCS‐12, and PCS‐12 showed significant improvement at the final follow‐up. The groups had no differences in postoperative patient‐reported outcomes at the final follow‐up, which suggest both procedures can achieve excellent clinical outcomes and improve the quality of life for patients. However, there were no significant improvements of the ODI, MCS‐12 and PCS‐12 in the LS‐PLIF group at 6 months after surgery. This finding indicated that patients in the LS‐PLIF group may encounter a problem of delayed rehabilitation postoperatively. In the follow‐up of HRQOLs such as ODI and SF‐12/36, there are controversies in different clinical studies. In a systematic review published by Zheng et al. [23], 13 studies focused on ADS treated by short segments fusion or long segments fusion were included. At the final follow‐up, the three results of higher ODI in the short segment group, higher ODI in the long segment group, or no difference between the two groups were present in all the research results. Zhang et al. found that longer fusion levels lead to high stiffness disability [24], which showed significantly worse scores in all HRQOLs such as ODI, JOA and SF‐36 compared with patients of low‐stiffness group, in the treatment of ADS. The research by Yoshida et al. indicated that in the surgical treatment of ADS, the inclusion of sacral or iliac fusion could slow down the decline in postoperative ODI scores and increase the difficulty of self‐care for postoperative patients [25]. Therefore, although after neural decompression and strong internal fixation, both short‐segment and long‐segment fusion procedures often obtain satisfactory VAS scores [26, 27], the improvement of postoperative HRQOLs is often affected by many complex factors. In the author's opinion, patients undergoing long‐segment PLIF have greater intraoperative trauma and longer hospital stays. After the operation, patients are more likely to return home rather than go to specialized rehabilitation institutions. These not only lead to a relatively heavy psychological burden, but also affect the efficacy of self‐rehabilitation, thereby prolonging the time for postoperative functional recovery.

**FIGURE 1:**
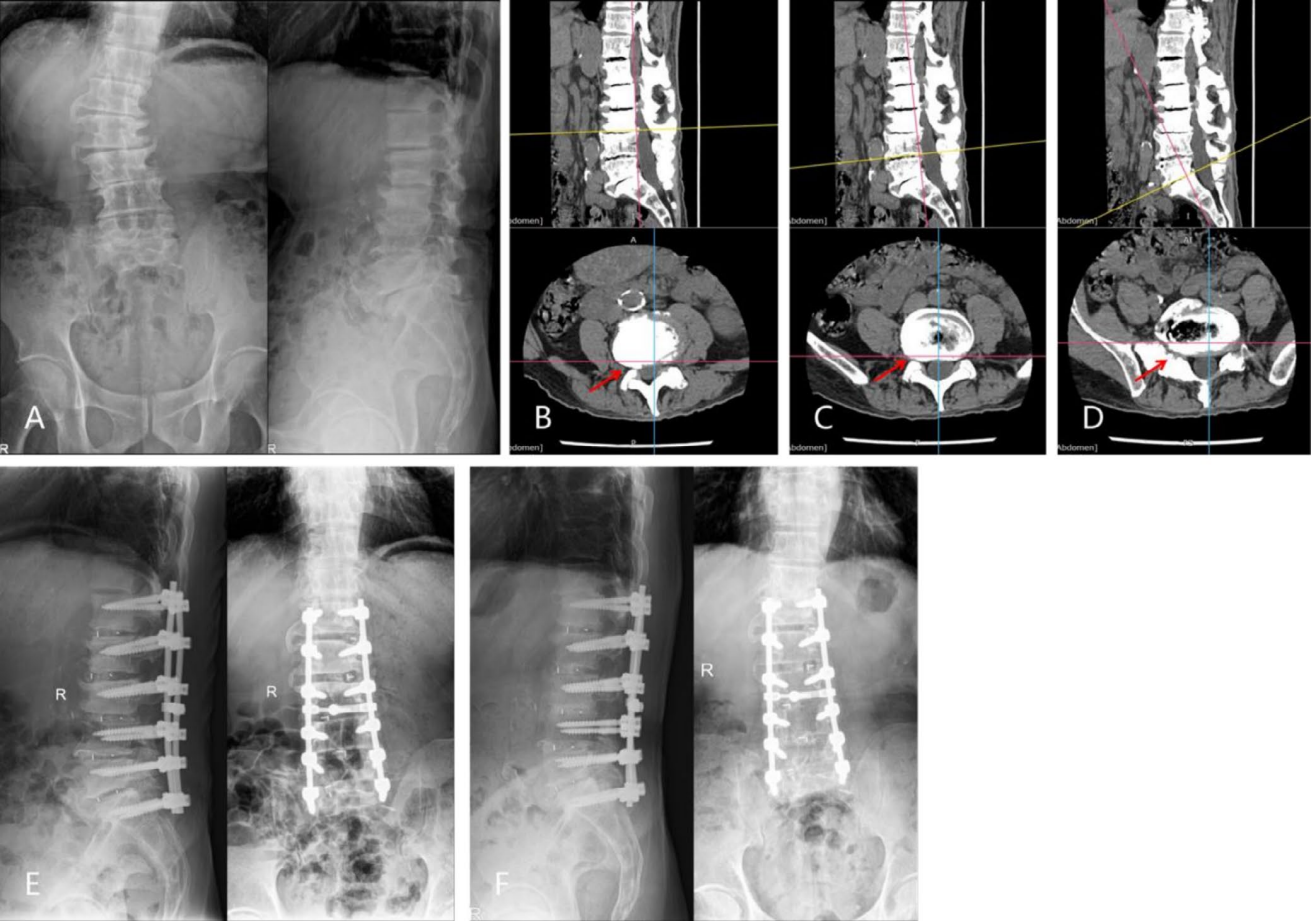
(A) Preoperative coronal and sagittal lumbar radiographs of a patient showing degenerative scoliosis. (B–D) Preoperative CT revealed multiple stenoses of intervertebral disk spaces and intervertebral foramina, most prominently manifested on the right side at the L3/4, L4/5, and L5/S1 levels. (E) The patient was treated with long‐segment PLIF; postoperative sagittal X‐ray revealed restoration of intervertebral disk height, with correction of the coronal Cobb angle. (F) Postoperative radiographs of the same patient taken 1 year postoperatively showing the correction of the Cobb angle and adjacent segment degeneration at the top.

There were significant improvements with PT, LL, and Cobb angles in the LS‐PLIF group. Previous studies have also offered recommendations for correction of severe coronal and sagittal imbalance by longer fusions which provided sufficient stability [3, 19]. For patients with proposed long‐segment PLIF, SVA, and LL play an important role in maintaining the overall balance of the spine and are closely related to postoperative HRQOLs. Surgical plans aiming to SVA reduction and LL restoration facilitate the achievement of optimal clinical outcomes [28]. However, long‐segment PLIF often implies a higher incidence of complications. Sagittal imbalance following long‐segment PLIF demands clinical vigilance, as it frequently precipitates distal complications including pseudarthrosis and hardware failure at the lumbosacral interface [29]. Furthermore, clinical evidence indicates that pedicle screw loosening frequently occurs in ADS patients undergoing long‐segment instrumentation, notably involving the caudal and cranial fusion levels [30]. In addition, adjacent segment degeneration (ASD) cannot be ignored in both short‐segment or long‐segment instrumented fusions. Rigid internal fixation enhances postoperative mobility and accelerates osseous consolidation, yet elevated stiffness in stabilized segments induces secondary mechanical stresses at adjacent spinal levels. Clinical biomechanical studies demonstrate that augmented segmental rigidity triggers compensatory kinematic alterations, with loads redistributing from multiple segments to adjacent levels [31]. ASD occurred in both groups, while the LS‐PLIF group demonstrated higher incidences of pseudoarthrosis and hardware complications requiring revision surgeries (Figure 1). There was one case in the SS‐PLIF+SNRB group undergoing revision surgery with deformity progression, which was not common according to previous studies. Lee et al. reported that the global magnitude of progression of the Cobb angle after short‐segment lumbar fusion surgery in patients with degenerative lumbar stenosis is similar to the natural curve progression of ADS [32]. In order to reduce the complications caused by long‐segment fusion, several studies have put forward their own viewpoints. Takami et al. demonstrated that strict adherence to “PI—LL mismatch < 10°” was not necessary in the elderly undergoing corrective fusion surgery for ADS [33]. Therefore, the number of fused segments and osteotomy would both decrease, thereby reducing the incidence of operative trauma and complications. Another study has demonstrated equivalent deformity correction and clinical outcomes between Oblique Lateral Interbody Fusion (OLIF) combined with posterior instrumentation and standalone posterior spinal fusion in degenerative scoliosis management [34]. The OLIF procedure features anterior placement of large interbody cages that expand intervertebral height, thereby achieving indirect spinal canal decompression while simultaneously restoring coronal/sagittal balance. Subsequent posterior instrumentation reduces fixation segments and minimizes iatrogenic trauma, synergistically enhancing therapeutic outcomes through optimized biomechanical stabilization [34, 35].

**FIGURE 2:**
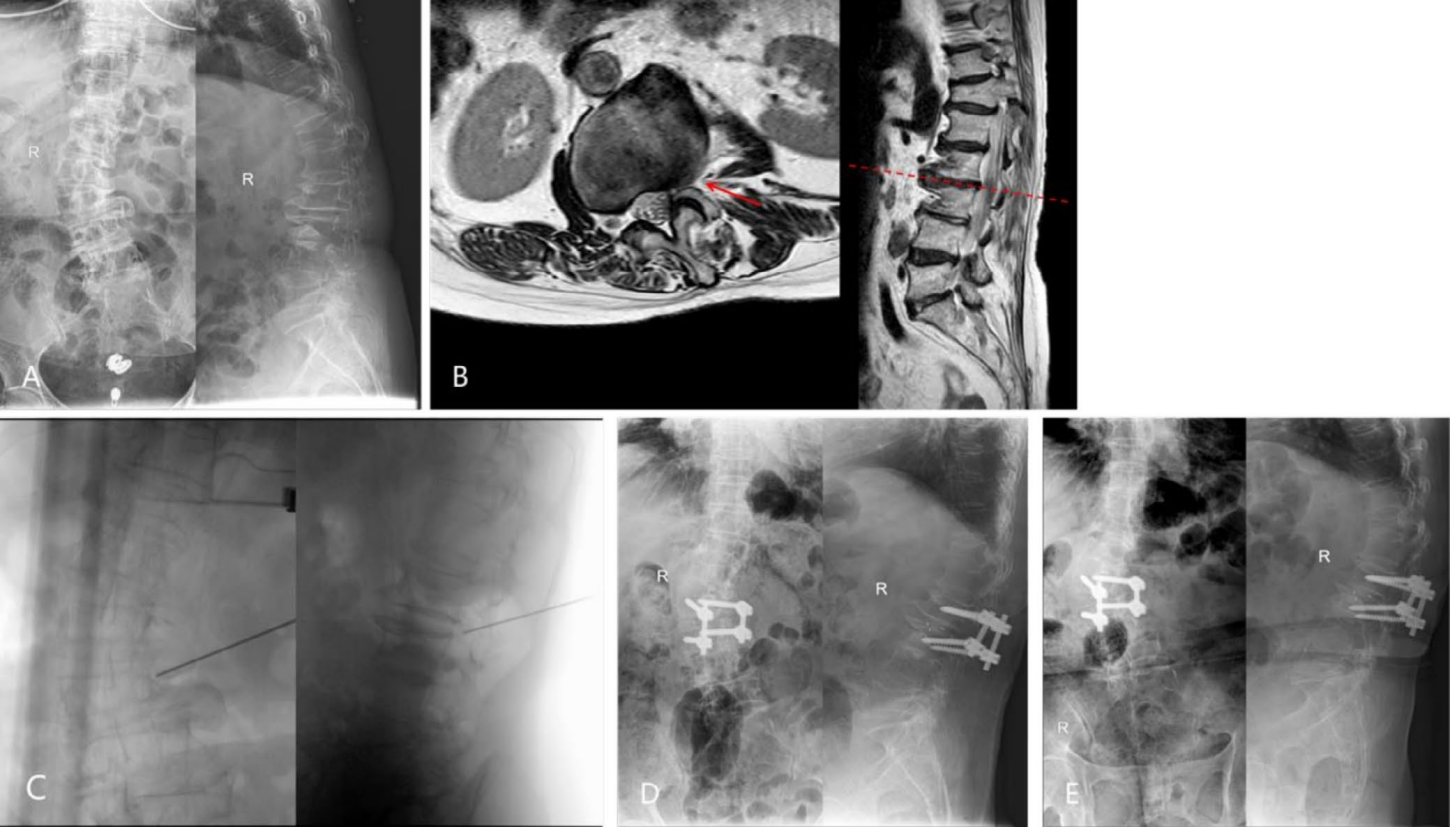
(A) Preoperative coronal and sagittal lumbar radiographs of a patient showing degenerative scoliosis. (B) Significant foraminal stenosis was observed on preoperative MRI. (C) A selective nerve root block was performed at L2/3 and achieved 80% relief of leg pain. (D) Then a single‐segment PLIF was performed the next day. (E) Postoperative radiographs of the same patient taken 6 months after surgery.

In our study, more patients presented radicular symptoms in the SS‐PLIF+SNRB group and had faster improvement in ODI, PCS‐12, and MCS‐12. It indicates that when mild degenerative scoliosis coexists with radicular symptoms, performing surgery at the pathologic level is sufficient to provide significant benefit in associated outcome domains (Figure 2). Before surgery, SNRB demonstrates prognostic utility in the single‐segment PLIF procedure by establishing a positive correlation between preoperative diagnostic blockade efficacy and postoperative neural decompression outcomes. This predictive capability enables surgeons to optimize surgical strategy selection while simultaneously enhancing patient compliance through validation of symptomatic nerve root involvement, reducing perioperative anxiety, and improving therapeutic confidence. The subgroup analysis further refines this view, positioning single‐segment PLIF with SNRB as a particularly advantageous option for patients whose primary complaint is radiculopathy, regardless of coronal deformity magnitude, while highlighting the continued role of long‐segment fusion for cases dominated by severe mechanical back pain and significant imbalance. Considering the risk, rehabilitation pathway, and costs of long‐segment radical surgery, short‐segment limited intervention is a better strategy for patients who cannot tolerate the long‐segment surgery, to improve symptoms and maintain efficacy in the mid‐ and long‐term, and not increase the reoperation rate [36, 37].

### 4.2 Optimizing SNRB Protocol for Surgical Planning

SNRB has both diagnostic and therapeutic values clinically. And there are relatively large differences in practical applications. For diagnostic purposes, SNRB emphasizes the precision of localization, strictly adhering to the exit root block rather than lesion block. There are three approaches to reach the exit root: sub‐pedicular, retro‐neural, and retro‐discal [38]. Several studies suggest the use of contrast agent to prevent the puncture needle from entering blood vessels and assess the blockage efficacy through different diffusion patterns [39]. In our view, the three puncture approaches described above are all designed to safely position the needle tip on the dorsal aspect of the exit root. Only when stimulation of the nerve root sheath with the needle tip reproduces pain in the same location as the patient's chief complaint, and subsequent nerve block alleviates pain consistent with the described chief complaint, can this intervention be considered truly diagnostically valuable. Additionally, multiple studies [40, 41, 42] have demonstrated that excessive injection doses of local anesthetics may inadvertently lead to adjacent segmental nerve root blockade or even epidural anesthesia spread. This phenomenon increases the false‐positive rate during diagnostic procedures, thereby compromising the accuracy of identifying the responsible spinal segment and ultimately rendering the intended diagnostic purpose ineffective. The criteria for determining a positive SNRB remain controversial, with the general standard for a positive SNRB being symptom relief of 50% to 100% after the block [16]. Therefore, in our study, we only administered 0.5 mL of 1% lidocaine without mixing with contrast agents or glucocorticoids and a VAS leg score improvement of more than 50% was considered a positive result. SNRB performed through Kambin's safe triangle for therapeutic purposes is safer and more effective.

It shares the same puncture technique with the transforaminal endoscopic surgery through the Kambin safe triangle, reducing complications such as nerve and vascular injuries. For patients with severe foraminal stenosis due to scoliosis, the puncture is safer and easier. However, as a regional block, it targets the traversing nerve root. If the puncture needle is positioned too deep or the dosage of blockage is excessive, it may lead to epidural or even intrathecal anesthesia, resulting in a less precise selective block and a higher false‐positive rate, though the therapeutic effect remains favorable.

### 4.3 Indications for Single‐Segment PLIF With SNRB

- A confirmed diagnosis of ADS (cobb angle > 10°).
- Progressive low back and leg pain, or refractory nerve root pain with neurological deficits (e.g., intermittent claudication) that severely impairs daily life and is unresponsive to conservative treatment.
- Radiological evidence (e.g., MRI, CT) of stenosis, but with symptoms that can be attributed to a single level, as confirmed by a positive selective nerve root block (SNRB) response (defined as > 50% pain relief).
- Absence of severe sagittal or coronal imbalance that necessitates long‐segment correction (e.g., SVA > 50 mm).

### 4.4 Limitations

This study has several limitations. First, its retrospective, single‐center design may introduce selection bias and limit the external validity of the findings.

Second, although patients with severe osteoporosis and marked sagittal imbalance were excluded and a subgroup analysis based on BMD was performed, the effects of moderate bone loss and mild sagittal malalignment were not fully evaluated. Therefore, the results are most applicable to carefully selected ADS patients without severe osteoporosis or significant sagittal imbalance.

Third, SNRB was used primarily as a diagnostic and surgical planning tool rather than a definitive long‐term therapeutic intervention, and its long‐term durability was beyond the scope of this study.

Finally, although a minimum follow‐up of more than 2 years was achieved, longer‐term outcomes beyond 5 years—particularly adjacent segment degeneration and deformity progression—require further investigation in future prospective studies.

## 5 Conclusion

Single‐segment PLIF with SNRB can achieve the same efficacy as a long‐segment PLIF for treating ADS. Single‐segment PLIF with SNRB in appropriately selected patients may provide satisfactory improvements in ODI, SF‐12, and VAS leg score, and mitigate hospital length of stay and revision surgery rates.

## Author Contributions

X.T., P.Y. contributed to the conception of the study. S.Z. performed the data collection, H.M. performed the data analysis and wrote the manuscript.

## Funding

The authors have nothing to report.

## Ethics Statement

The study was approved by the ethics committee of China‐Japan Friendship Hospital. Signing the informed consent was waived by the ethics committee of China–Japan Friendship Hospital.

## Consent

Written informed consent for publication of their clinical details and clinical images was obtained from the patient/parent/guardian/relative of the patient.

## Conflicts of Interest

The authors declare no conflicts of interest.

## Data Availability

The data that support the findings of this study are available from the corresponding author upon reasonable request.
